# Self-tolerance in multiple sclerosis

**DOI:** 10.1007/s13760-012-0061-x

**Published:** 2012-03-24

**Authors:** R. E. Gonsette

**Affiliations:** Fondation-Charcot-Stichting, Avenue Huart Hamoir 48, 1030 Brussels, Belgium

**Keywords:** Multiple sclerosis, Self-tolerance, Autoimmunity, Regulatory T cells, T cell homeostasis

## Abstract

During the last decade, several defects in self-tolerance have been identified in multiple sclerosis. Dysfunction in central tolerance leads to the thymic output of antigen-specific T cells with T cell receptor alterations favouring autoimmune reactions. In addition, premature thymic involution results in a reduced export of naïve regulatory T cells, the fully suppressive clone. Alterations in peripheral tolerance concern costimulatory molecules as well as transcriptional and epigenetic mechanisms. Recent data underline the key role of regulatory T cells that suppress Th1 and Th17 effector cell responses and whose immunosuppressive activity is impaired in patients with multiple sclerosis. Those recent observations suggest that a defect in self-tolerance homeostasis might be the primary mover of multiple sclerosis leading to subsequent immune attacks, inflammation and neurodegeneration. The concept of multiple sclerosis as a consequence of the failure of central and peripheral tolerance mechanisms to maintain a self-tolerance state, particularly of regulatory T cells, may have therapeutic implications. Restoring normal thymic output and suppressive functions of regulatory T cells appears an appealing approach. Regulatory T cells suppress the general local immune response via bystander effects rather than through individual antigen-specific responses. Interestingly, the beneficial effects of currently approved immunomodulators (interferons β and glatiramer acetate) are associated with a restored regulatory T cell homeostasis. However, the feedback regulation between Th1 and Th17 effector cells and regulatory T cells is not so simple and tolerogenic mechanisms also involve other regulatory cells such as B cells, dendritic cells and CD56^bright^ natural killer cells.

## Introduction

It is somewhat controversial as to whether multiple sclerosis (MS) is primarily an autoimmune disease. One of the defining features of autoimmune diseases is the failure of the immune system to maintain self-tolerance against tissue-specific antigens (TSAs). MS shares physiopathologic mechanisms with other autoimmune disorders, notably a genetic predisposition modulated by epigenetic and environmental factors. Some of those risk factors are common to several autoimmune diseases. It has even been suggested that the interactions between genetic, epigenetic and environmental risk factors might determine the clinical phenotype of the autoimmune disorder [1]. The mechanisms responsible for maintaining or restoring self-tolerance have been the focus of significant attention during the last decade. In this review, we will concentrate on recent data concerning the breaches in central and peripheral tolerance in MS as well as their potential therapeutic implications.

## Central tolerance

Central tolerance results from the elimination of autoreactive cells in the thymus for T cells and bone marrow for B cells, leading to the generation of mature T and B cell repertoires that recognize exogenous pathogens while displaying tolerance to self-antigens.

Positive selection tests the ability of the T cell receptor (TCR) to signal in response to self-peptide-major histocompatibility (MHC) complexes displayed by cortical thymic epithelial cells (cTECs). Thymocytes-expressing TCRs with very low or no affinity for MHC complexes, die by neglect. Surviving thymocytes pass subsequently in the thymic medulla where they encounter medullary thymic epithelial cells (mTEC) that express TSAs [2]. Negative selection eliminates thymocytes with a strong signal in response to self-MHC-peptide via active apoptosis.

Negative selection is mainly mediated by the autoimmune regulator (Aire) gene encoding the Aire protein [3] that modulates the intracellular expression of TSAs. Recently, Aire has also been identified in peripheral lymphoid organs where it regulates expression of TSAs distinct from those expressed in the thymus [4]. Interestingly, transplantation of Aire-encoding bone marrow cells in mice attenuates the development of MOG-induced experimental autoimmune encephalomyelitis (EAE) [5].

Protection against autoimmunity for a specific organ requires a minimal expression threshold level of the concerned TSA. Thymocytes that receive TCR signals just below the threshold for negative selection can undergo a program of differentiation to become natural CD4^+^CD25^+^Foxp3^+^ regulatory T cells (nTreg) [6]. Importantly, both mTECs and dendritic cells (DC) cells contribute to negative selection of naïve T cells and generation of nTregs [7].

The presence of autoreactive T cells in the periphery in MS patients and in healthy controls (HC) demonstrates that central tolerance is not completely efficient. Escape from negative selection may result from TCR dysfunction or from a weak binding of the self-peptide to the MHC II, which destabilizes the complex with TCR.

Alterations in the crystal structure of TCRs from two MS patients have been identified [8]. Both TCRs engage peptide–MHC complex through altered docking modes. The TCR is centred not over the central portion of the MBP–MHC complex, but over its N-terminal portion. Consequently, the overall stability of the TCR–MBP–MHC complex is markedly reduced, its affinity is suboptimal and the thymocytes avoid negative selection. Recently, another structural analysis of the TCR from a MS patient has been reported [9]. In this patient, the TCR engaged the MBP–MHC complex in a canonical mode and had a strong affinity for this complex, but the binding of MBP to HLA-DR4 was unusually weak and permitted escape from negative selection.

Another aspect of thymic dysfunction in MS is its inability to maintain T cell homeostasis. A defect in global naïve T cell thymic output, as demonstrated by a marker of newly exported naïve T cells (TCR excision circles-TREC), has been demonstrated in young relapsing–remitting (RR) MS patients [10]. Further studies showed that TREC numbers in MS patients were lower than those in age-matched HC suggesting a premature thymic involution in MS [11, 12]. TRECs decrease significantly with increasing age in HC whereas no age-associated TREC reduction is observed in MS.

This particular thymic dysfunction in MS was confirmed with CD31^high^, a marker for recent, immature CD4 thymic emigrant (RTEs) naïve T cells [13]. Similar thymic alterations, even more pronounced, have been reported in patients with primary progressive (PP) MS. Those observations suggest a low thymic export early in life and likely before disease onset [14].

## Peripheral tolerance

Peripheral tolerance is regulated by complex T cell intrinsic mechanisms (costimulatory signalling, transcriptional and epigenetic mechanisms) as well as by extrinsic mechanisms (regulatory T cells).

### Intrinsic mechanisms

#### Costimulatory molecules

In addition to the TCR–peptide–MHC complex, costimulatory signals are required to activate T cells. In the absence of these signals, T cells become “anergic” and induce self-tolerance.

**CD28** binds to B7.1 (CD80) and B7.2 (CD86). CD4^+^ CD28^null^ cells have been identified in peripheral blood of MS patients. Less sensitive to regulatory mechanisms, they have indirect pathogenic capacities [15] and were found to produce higher levels of IFNγ [16].

**ICOS** (inducible costimulator protein) binds to B7H and modulates Th1/Th2 cytokine production. Genetic analyses show that the frequency of ICOS gene AA homozygosity is significantly lower in patients with RR MS than in HC. Patients expressing the AA homozygosity have lower relapse rate and disability score [17].

**CD40** plays an ambivalent role in MS pathogenesis [18]. Engagement of CD40 with its ligand (CD40-L = CD154) in mTECs can contribute to the development of central tolerance. On the other hand, CD40 promotes T cell differentiation to Th17 cells. In MS patient brain lesions, CD40-L is expressed on activated CD4 cells in secondary progressive (SP), but not in RR MS or in HC [19]. Its higher expression on peripheral lymphocytes in SP patients might be associated with the shift from RR to SP MS [20, 21].

**CTLA-4** (cytotoxic T-lymphocyte Antigen 4) binds to the same receptors as CD28 but has a negative regulatory function. CTLA-4 expression is lower in SP than in RR MS [22] and a low percentage of cells expressing CTLA-4 seems associated to earlier transition to the SP stage [23].

**PD-1** (programmed death pathway 1) is expressed by activated T cells. A strong upregulation of its ligand PD-L1 (also known as B7H1) has been observed in MS lesions and identified as a down-regulator of T cell responses [24]. A significant association of a PD-1 polymorphism with the progressive form of MS has been reported [25].

**4-1BB** (CD137) expression is downregulated on both Tregs and DC in MS [26, 27]. The soluble form of its ligand (s4-1BBL) is consistently elevated in plasma [28] and cerebrospinal fluid (CSF) [29] of MS patients. Those observations suggest that altered 4-1BB/s4-1BBL interactions may be involved in the impaired regulatory function of Treg and plasmacytoid DC in MS patients.

**CD58** mRNA expression is significantly increased in MS patients during relapses. Its protective effect seems partly due to an enhancement of Foxp3 expression in Tregs [30].

#### Transcriptional mechanisms

Foxp3, a transcriptional factor responsible for the differentiation of CD4 and CD8 cells in the regulatory phenotype, is impaired in RR MS [31]. Altered apoptotic mechanisms have been observed in RR MS [32] and microarray analyses have identified a dysfunction of nuclear receptor family members in the pre-disease state of MS [33]. Both mRNA and protein levels of Casitas B cell lymphoma-b (Cbl-b), a negative regulator of T cell activation, inversely correlate with relapses and are significantly decreased in MS patients particularly during exacerbations [34].

#### Epigenetic mechanisms

Several observations suggest that histone modifications are involved in programming T cell tolerance. Tolerant CD4 and CD8 T cells exhibit threefold more DNA methylation within IL-2 promoter compared with activated cells [35]. Epigenetic mechanisms control the fate of Tregs by inducing and stabilizing Foxp3 expression [36].

### Extrinsic mechanisms

This review will be limited to regulatory T cells, excluding regulatory B cells, tolerogenic DC and CD56^bright^ NK cells. Suppressive activity of T cells received little attention till the observation that self-tolerance was maintained by activated CD4 cells expressing IL-2 receptor (CD25) in mice [37] and in humans [38]. Progress related to phenotype and function of T cell subsets made possible the identification of CD8 regulatory T cells in rats [39] and in MS patients [40].

#### CD4^+^CD25^+^Foxp3 regulatory T cells (Tregs)

CD4^+^CD25^+^Foxp3 cells represent a heterogeneous population and the lack of a specific marker for the Treg cell phenotype likely explains some contrasting observations concerning their frequency and function.

In addition to CD25, Foxp3 is a master regulatory gene for their cell-lineage differentiation and determines their commitment in the maintenance of immune homeostasis and self-tolerance. The Foxp3 protein expression at the cellular level correlates with the suppressive activity of Tregs [31].

Natural Tregs are functionally mature antigens primed in the thymus before encountering antigens in the periphery and competent to prevent autoimmunity [41]. They migrate to peripheral secondary lymphoid organs. Within lymph nodes, they interact with DC and block their capacity to prime naïve CD4 and their subsequent differentiation into autoreactive self-specific T cells [42]. They exert their suppressive activity by cell-to-cell contact.

According to specific modes of antigen stimulation in a particular milieu, Foxp3 can convert naïve CD4 T cells into the inducible regulatory phenotype (iTregs) functionally similar to nTregs [43]. They only differ in their epigenetic regulation of Foxp3 [44]. Their suppressive effects do not require cell-to-cell contact but are mainly mediated by cytokines such as IL-10 and IL-35 as well as by TGFβ [45]. Importantly, uncommitted nTregs retain developmental plasticity and both fully differentiated iTregs and nTregs can be reprogrammed by IL-6 towards the Th17 lineage [46–48].

The nature of many suppressive mechanisms exerted by Tregs remains elusive and it remains unclear whether they use the same or different mechanisms to control different cell types. Treg activation requires antigen specific recognition but Treg suppression is non-specific and is exerted via bystander effects.

Observations concerning the frequency of nTregs in the periphery between MS patients and HC are contradictory. No differences [49–52] as well as an increase in their numbers [53, 54] has been reported.

In contrast, the numbers of Tregs are consistently found increased in the CSF of MS patients compared with HC [52, 55]. Of note that Tregs cells are scarce in MS brain lesions [56] whereas in EAE, they proliferate into the CNS [57].

A significant defect in the suppressive function of nTregs was first demonstrated in RR MS [49] and was further confirmed by a lower inhibitory effect of Tregs from MS patients on Ag-specific T cell proliferation induced by recombinant MOG and allogenic stimuli [51]. An altered suppressor function of Tregs associated with a low mRNA expression of Foxp3 was observed in RR, mainly in the early phase of the disease, but not in patients with SP MS who have normal suppressive function and normal Foxp3 expression [31]. The defect in immunosuppressive activity actually lies in the Treg function as CD4 responder cells were not refractory to suppression [49, 51].

A genome-wide expression analysis in Tregs from MS patients shows that 23 miRNAs are differentially expressed compared with HC. Down-regulation of TGFβ pathways by miRNAs might result in altered Treg function [58].

Other functional surface markers that are components of the suppressive mechanisms of Tregs have also been investigated. A subset of Tregs bearing the CD39 and CD73 markers seems more specifically involved in regulating IL-17 toxicity suppression [59]. Both CD39 and CD73 are potent immunosuppressive molecules. Patients with RR MS have markedly reduced numbers of CD39 in the blood [60]. Their suppressive activity is less effective in RR compared with SP MS.

Tregs express low levels of CD127 (IL-7 receptor α chain), a marker of activation that negatively correlates with Foxp3 expression and suppressive activity. When CD4^+^CD25^+^CD127^low^ Tregs were segregated in naïve and memory T cells, the suppressive activity of naïve Tregs after CD3 costimulation was found reduced in both RR and SP MS. Of note that memory Treg numbers and function were restored during the progressive phase [61]. More recently mature and non-mature CD127^low^ Tregs were found defective in suppressive activity after CD2 costimulation in patients with RR MS [62]. In contrast, using a more stringent threshold for CD25 expression and after elimination of CD127^high^ Tregs, remaining Tregs had normal function in RR MS patients [63]. This underlines the difficulty to accurately identify Tregs in the absence of a specific marker.

In non-inflammatory conditions, Tregs participate to immunosurveillance. They express the CCR6 adhesion molecule and in HC exhibit a stronger migratory response to CCL20 than Th17 cells. In RR MS, their migratory capacities are significantly impaired, facilitating the initiation of inflammation by Th17 cells [64].

An increase in percentage of CD4^+^CD25^+^ correlating with disease activity was first reported in 2000 [65]. An evaluation of Treg markers in stable and relapsing patients has been recently performed with usual markers of Tregs (CD25 and Foxp3) as well as “functional” markers (CD39/CD73, CTLA-4 and glucocorticoid-induced TNF-GITR) that are supposed to fluctuate with disease evolution [66]. In patients longitudinally followed for 1 year, whatever the expressed markers, strikingly reduced numbers of Tregs were found in stable patients compared with HC. In contrast, all Treg subset numbers and suppressive function were significantly increased during relapses.

#### CD8^+^CD25^+^Foxp3^+^ (CD8 Tregs)

After a decade of focusing on CD4 Tregs, CD8 Tregs have recently gained interest [67]. Like their CD4 counterpart, CD8 Tregs are a heterogeneous population. There is no specific marker of CD8 Tregs. Functional markers categorize them as natural (from thymic output) or induced (from the post-thymic cell pool). CD8 Treg cells can be induced from CD8^+^CD25^−^ T cells by continuous antigen stimulation. Several studies have shown that CD8 Tregs recognize and lyse activated myelin-specific CD4 cells [68]. This cytotoxic response is decreased in peripheral blood and in the CSF in MS patients during exacerbations [40, 69]. Apart from exacerbation periods, numbers and suppressive function of CD8 Tregs do not seem to differ between MS patients and HC [70].

#### Other regulatory T cells

**Tr1** is a regulatory T cell phenotype characterized by a high amount of IL-10 secretion. A marked defect in the induction of Tr1 cells has been observed in patients with MS compared with HC [71].

**Th3** regulatory T cells producing TGFβ are generated in MS patients after oral administration of myelin antigens [72].

**HLA-G** regulatory T cells are CD25^−^ Foxp3^−^ cells exerting their suppressive activity independently of IL-10 and IFNβ [73]. A correlation with clinical and MRI disease activity for sHLA-G CSF levels [74] but not for serum levels [75] has been reported.

**TCR-αβ CD4** ^−^ **CD8** ^−^ double negative T cells (DN-T) highly differ from nTreg [76]. They do not express the marker Foxp3 and exert their suppressive activity by modulating TCR signalling. The immunosuppressive properties of DN-T have not been explored in EAE or MS so far.

## Discussion

Multiple sclerosis is characterized by three closely intermingled, basic pathomechanisms: autoimmune reactions, non-resolving inflammation and neurodegeneration. Non-resolving inflammation can initiate autoimmunity and conversely autoimmunity can initiate inflammation. Both immune reactions and inflammation can mediate neurodegeneration but which comes first in MS remains an open question.

Several defects in self-tolerance have been recently identified in MS. Dysfunction in central tolerance leads to the thymic output of antigen-specific T cells with TCR alterations favouring autoimmune reactions [8, 9]. Moreover premature thymic involution in MS results in a reduced export of RTE naïve Treg, the fully suppressive Treg clone [13]. The low thymic output of naïve Tregs cannot maintain a functional homeostasis despite a stable cell count of the total Treg population due to an increased proliferation of less immunosuppressive memory Tregs. Peripheral Treg cell development alone is not sufficient to establish self-tolerance and generation of Treg cells in the thymus is crucial for maintaining immune homeostasis. Some observations argue firmly for the presence of those self-tolerance defects before the outset of the disease [14, 33].

Dysfunctions of peripheral tolerance concern costimulatory molecules as well as transcriptional and epigenetic mechanisms. Recent data underline the key role of Tregs that suppress Th1 and Th17 effector cell (Teff) responses. Teff are the main source of IL-2 and, on the other hand, IL-2 is essential for Treg expansion. It has been proposed that this interdependence constitutes a feedback mechanism, impaired in MS patients, indexing the number of Treg cells to the number of Teff cells and adapting their respective proportions to maintain a Teff/Treg equilibrium and immune system homeostasis [77].

Those recent observations suggest that a defect in physiological mechanisms, whose purpose is to prevent or to regulate and terminate a potential response to circulating myelin specific T cells, might be the primary cause of MS leading to immune reactions and subsequent inflammation. In line with this idea, the genetic predisposition of MS notably concerns mechanisms regulating autoimmune mediated inflammatory responses [78] and determining individual’s inherent ability to resist autoimmune induction [79].

No major differences between circulating myelin specific cells in the blood of MS patients and healthy controls have been observed. However, the last mentioned do not develop MS. Furthermore, if a local tissue injury in the CNS (e.g. viral infection) leading to myelin destruction was the prime mover of autoimmune reactions and subsequent inflammation it seems difficult to understand why patients with stroke or brain trauma do not develop progressive demyelinating lesions. In those patients indeed, myelin destruction leaks brain specific antigens that are exposed to immune reactive cells and can be measured in the CSF [80]. Even if the conditions are present, autoimmune reactions do not develop in the CNS. It seems therefore that, in contrast to MS patients whose regulatory mechanisms are compromised, patients with a healthy immune system are able to control and terminate inflammatory damages when myelin specific T cells accidentally recognize exposed brain antigens (CNS injury-induced immunodepression syndrome, CIDS). Also noteworthy is the fact that Tregs counteract production of proinflammatory cytokines, lymphocyte infiltration and microglial activation via IL-10 production in experimental acute stroke [81].

The concept of MS as a consequence of the failure of central and peripheral tolerance mechanisms, particularly of Tregs, to maintain a self-tolerance state may have therapeutic implications. Restoring normal Treg function appears an appealing approach. Indeed, Tregs globally suppress local responses via bystander effects rather than through a specific response for the antigen recognized by Tregs. Of particular interest are the observations that the beneficial effects of currently approved immunomodulators are associated with a restored Treg homeostasis. Administration of GA stimulates the differentiation of CD4^+^CD25^−^ into iTregs [82] and activates the thymopoietic pathways [83] restoring the balance between naïve Tregs and memory Tregs [84]. The same holds true for IFNβ and INFα treatments that restore and/or increase Treg populations and function directly or via DC modulation [54, 55, 85–87].

Numerous approaches in experimental models of autoimmune diseases are currently investigated to boost Tregs numbers and function. Adoptive cell transfer of patient-specific Tregs requires overcoming technical problems and would be quite expensive. Anti-CD3 therapy causes a dramatic expansion of a specific and strongly immunosuppressive Treg population restricted to peripheral “niches” in lymphoid organ [88]. Short-term low-dose IL-2 treatment preferentially targets Tregs rather than Teff cells and induces a long-term protection in experimental type 1 diabetes [89]. Treg cell epitopes (tregitopes) are peptides expressed in the Fc and Fab domains of IgGs. They specifically induce and expand iTregs and generate antigen-specific adaptive tolerance to MOG epitopes in vivo [90]. We must not forget that the translation of experimental benefit into therapies for human patients can lead to unexpected adverse outcomes. Selective targeting of Tregs with superagonistic anti-CD28 antibodies induces a marked Treg expansion and protects from EAE [91]. Well tolerated in animals, its infusion into healthy people in a phase I trial provoked a life-threatening cytokine storm [92].

## Conclusion

During the last decade, numerous observations have underscored the failure to maintain self-tolerance as an important pathomechanism in MS development and evolution. Alterations of regulatory T cells as early as from their generation in the thymus strongly support the concept of MS as an autoimmune disease. MS seems thus to be primarily the result of the failure to sustain protective immunity [79]. In MS, Tregs cannot prevent circulating specific T cell activation and/or terminate subsequent inflammatory reactions. This Treg impairment gives rise to non-resolving inflammation during which acute and chronic inflammation may coexist. Inflammation then initiates a degenerative cascade and tissue injury. At the moment, our current treatments can only target inflammatory processes. Our recent understanding of immune mechanisms initiating inflammatory and degenerative cascades holds the potential to restore the altered self-tolerance associated with MS pathogenesis. It should be noted, however, that the Teff/Treg homeostasis concept is not so simple and that regulatory mechanisms involve other tolerogenic cells such as B, DC and CD56^bright^ NK cells.
